# Gay’s Anti-Typhoid Vaccine

**Published:** 1915-11

**Authors:** 


**Gay's Anti-Typhoid Vaccine.** Report of F. P. Gay of Univ. of Cal. to Am. Soc. of Trop. Med. "A sensitized vaccine sediment" is used therapeutically. Abrupt cures are reported in 5 of 14 cases.

